# Implementing patient-centred outcome measures in palliative care clinical practice for adults (IMPCOM): Protocol for an update systematic review of facilitators and barriers

**DOI:** 10.12688/f1000research.131479.2

**Published:** 2023-10-30

**Authors:** Bárbara Antunes, Stephen Barclay, Isla Kuhn, Kathy Eagar, Claudia Bausewein, Fliss Murtagh, Simon Etkind, Ben Bowers, Sarah Dixon, Roberta Lovick, Richard Harding, Irene Higginson, Farhad Shokraneh

**Affiliations:** 1 Palliative and End of Life Care Group in Cambridge (PELiCam), Department of Public Health and Primary Care, Primary Care Unit, The University of Cambridge, Cambridge, UK; 2 Medical Library, School of Clinical Medicine, The University of Cambridge, Cambridge, UK; 3 The Australian Health Services Research Institute, The University of Wollongong, Wollongong, Australia; 4 Department of Palliative Medicine, LMU Munich, Munich University Hospital, Munich, Germany; 5 Wolfson Palliative Care Research Centre, University of Hull, Hull, UK; 6 Department of Palliative Care and Cicely Saunders Institute, King's College London, London, UK; 7 Department of Evidence Synthesis, Systematic Review Consultants LTD, Nottingham, UK

**Keywords:** Patient-centred outcome measurement; patient-reported outcome measurement; palliative care; systematic reviews; implementation; barriers; facilitators

## Abstract

**Background**: Despite the development of patient-centred or patient-reported outcome measures (PCOMs or PROMs) in palliative and end-of-life care over recent years, their routine use in practice faces continuing challenges.

**Objective**: To update a highly cited literature review, identify and synthesise new evidence on facilitators, barriers, lessons learned, PCOMs used, models of implementation, implementation outcomes, costs, and consequences of implementing PCOMs in palliative care clinical practice.

**Methods**: We will search MEDLINE, PsycINFO, CINAHL, Embase, Emcare, SCI-Expanded, SSCI, ESCI, and BNI. The database search will be supplemented by a list of studies from the expert advisory committee, hand-searching of reference lists for included articles, and citations of the original review. We will include primary studies using a PCOM during clinical care of adult patients with advanced disease in palliative care settings and extract data on reported models of implementation, PCOMs, facilitators, barriers, lessons learned, costs, and implementation outcomes. Gough’s Weight of Evidence Framework will be used to assess the robustness and relevance of the studies. We will narratively synthesise and tabulate the findings. This review will follow PRISMA, PRISMA-Abstract, PRISMA-P, and PRISMA-Search as the reporting guidelines.

**Source of funding**: Marie Curie. The funder is not involved in designing or conducting this study.

**Protocol registration**: CRD42023398653 (13/02/2023)

## Introduction

The importance of using outcome measures to understand the effect and effectiveness of health interventions has been growing as they are increasingly credited as an essential component of evidence-based clinical practice, specifically, outcome measures that the patient completes - so-called patient-reported outcome measures or PROMs - because of their specificity to patients’ individual needs. Data collected at the individual patient level can be used immediately for patient-centred care, as it allows healthcare professionals to act on any distressing symptoms or palliative needs they might have. It can also inform shared decision-making and advance care planning ( Antunes *et al*. 2014, 2022; Antunes and Ferreira 2020). Additionally, PROMs data can be aggregated for audit, research, quality improvement and benchmarking ( Currow *et al*. 2015). However important the PROMs are, measuring can be challenging when the patient is becoming too ill or approaching the end of their life.

In palliative and end-of-life care, it is equally important to learn about physical effects and psychological, existential, emotional and practical outcomes. However, implementing PROMs as a routine part of palliative care clinical practice has been slow and challenging in a number of countries. Compared to other settings and conditions, measuring outcomes in palliative care faces unique challenges. One reason is that patients’ health is expected to deteriorate, and symptoms may worsen; deterioration will make the outcome measurement challenging, as well as changes in cognitive abilities towards the end of life. PROMs are impossible to use closer to the time of death once the patient becomes unable to communicate ( Bausewein *et al*. 2011). This raises ethical and practical challenges when proxy outcome measurements are used for patients with cognitive impairments ( Martins Pereira and Hernández-Marrero 2018).

Our previous review summarised barriers, facilitators, and lessons learned from the published palliative care literature and provided recommendations for implementing outcome measures at the patient, healthcare professional, and management and policy-makers levels for three timepoints: preparation, implementation, and assessment/improvement ( Antunes *et al*. 2014).

Our new systematic review will update and expand the previous review conducted in 2013 on facilitators, barriers, and lessons learned in implementing PROMs in palliative care clinical practice. In addition, we will seek to identify the implementation models used and the costs of implementing those measures. We will also review the literature concerning patient-centred outcome measures reported by family members and healthcare professionals when the patient is not able to do so themselves. For this review, we will use the term ‘patient-centred outcome measures’ (PCOMs) to include both patient-reported and proxy-reported measures that focus on the concerns important to patients.

### The need for an update

The original systematic review (cited 307 times on 16 January 2023) was published in *Palliative Medicine* and included the literature published between 1985 and 2013. In the last decade, there has been a plethora of publications demonstrating the exponential growth in palliative care outcome measurement. An update on these developments is warranted.

### Objectives

1. To identify the patient-centred outcome measures implemented in palliative care clinical practice;
2. To identify the facilitators of implementing patient-centred outcome measures in palliative care clinical practice;
3. To identify the barriers to implementing patient-centred outcome measures in palliative care clinical practice;
4. To identify lessons learned on implementing patient-centred outcome measures in palliative care clinical practice;
5. To identify the implementation models used when implementing patient-centred outcome measures in palliative care clinical practice;
6. To identify what implementation outcomes were measured and how, when implementing patient-centred outcome measures in palliative care clinical practice;
7. To assess the financial costs of implementing patient-centred outcome measures in palliative care clinical practice;
8. To update the recommendations from our previous literature to inform the implementation process in palliative care clinical practice for stakeholders at different levels.

## Methods

### Design

This systematic literature review and narrative synthesis was reported based on PRISMA-P ( Moher *et al*. 2015), and the final report of this review will be reported following PRISMA reporting guidelines, including PRISMA, PRISMA Abstract ( Page *et al.* 2021), and PRISMA-Search ( Rethlefsen *et al*. 2021). This protocol was registered on PROSPERO (CRD42023398653) on 13 ^th^ February 2023.

### Criteria for considering studies for this review

***Types of studies***

Primary research studies reporting information related to one of the eight objectives of this review outlined above. All types of evidence synthesis will be considered research studies and included.

***Types of participants***

Adult patients (18 years old and over) with advanced diseases and receiving palliative and end-of-life care, their informal carers, and healthcare professionals.

***Types of setting***

All settings in which palliative care is provided. As in the original review, we will include studies conducted both in specialist and non-specialist palliative care settings, provided that in the non-specialist settings, a palliative care measure was implemented.

***Implementation of outcome measures as the intervention***

The included articles will report on the implementation of PROMs. PROMs are a form of outcome measure and comprise standardised, validated questionnaires that patients complete to measure their perceptions of their own health status and well-being ( Etkind *et al*. 2015). Since focusing on PROMs alone runs the risk of excluding less well or unwell patients who may not be able to complete those measures themselves, proxy outcome measures completed by families and professionals - PCOMs - are useful and will be considered in this review. Previous authors have highlighted patient-centeredness as key to outcome measurement in palliative care ( Etkind *et al*. 2015; Anhang Price and Elliott 2018).

### Exclusion criteria

***Types of studies***

Narrative reviews, editorials, commentaries, and case reports will be excluded. If we identify relevant narrative reviews, we will check their references for eligible studies.

***Types of participants***

Studies with a paediatric or mixed population where fewer than 50% of participants are 18 years old or over.

***Types of setting***

Studies with non-palliative care settings or mixed settings when fewer than 50% of participants are from palliative care settings.

***Implementation of outcome measures as the intervention***

Studies reporting data from routine outcome measurement without information on the implementation process as they relate to settings in which implementation has already taken place.

Studies reporting exclusively on the development, testing and feasibility phases of new measures or validation of the linguistic translation of measures, as they do not relate to implementing measures in clinical practice.

### Search methods for identification of studies

***Electronic searches***

An information specialist will systematically search MEDLINE, Embase, and Emcare via Ovid SP, CINAHL and PsycINFO via EBSCOhost, British Nursing Index via ProQuest, Science Citation Index-Expanded, Social Science Citation Index, and Emerging Sources Citation Index via Web of Science without any limitations to language, date, document type, or publication status.

The searches were designed by an expert information scientist and peer-reviewed by another information scientist in collaboration with two clinical experts. The search strategies were tailored based on the included studies in the original review and tested against a new set of relevant studies supplied by the experts in MEDLINE before the agreement on the final search strategy.

The search strategy for MEDLINE via Ovid SP can be found as *Extended data* ( Shokraneh *et al.* 2023a).

***Searching other resources***

Citations of the original review in Web of Science, Google Scholar, and Scopus will be collected and screened. In addition, we will hand-search the reference lists of all included articles and relevant review articles.

### Study selection

The results will be imported into EndNote and de-duplicated. We expect to screen in the region of 20,000 records after removing duplicate records. The de-duplicated search results will be exported from EndNote and imported into Rayyan ( Ouzzani *et al*. 2016). Two reviewers will screen 20% of the results independently using the “blinding mode”. If there is more than 90% agreement, a single reviewer will screen each result after this point. Any discrepancies between the two reviewers will be resolved by discussion. All full reports will be independently checked by two reviewers. If a disagreement between the two reviewers continues, a senior topic expert will make the final decision on the eligibility of the studies.

As in the initial systematic review, we anticipate considerable heterogeneity of papers and that eligibility criteria would need to be supplemented with decision rules. These will be formalised and applied consistently; disagreements between research team members will be resolved by discussion.

### Data extraction and synthesis

The data extraction form will be designed, piloted and revised based on the following data points:

- Patient-centred outcome measures used (name, frequency, format, length)
- Outcome reporter (patient, nurse, family member, carer, general practitioner)
- Factors (facilitators, barriers) related to the implementation
- Implementation models used
- Outcomes of implementation ( Peters *et al*. 2013)
- Costs associated with implementation
- Type of implementation research objective ( Peters *et al*. 2013)
- Implications, proposals, or suggestions (lessons learned)
- Country
- Date of research and publication
- Age groups
- Healthcare conditions (cancer, non-cancer, etc.)
- Setting (home, hospice, hospital, palliative care unit, care home, etc.)
- Scale of the study (pilot, local or national studies of implementation)
- Study design
- Objectives of the study
- Limitations and strengths of the study
- Sample size
- Notes (including relevant information not matching other data points)
- Potentially relevant references
- Lead author's name and email address

We will initially use the form in Google Docs document format for piloting, and when finalised, we will use Google Sheets to extract data.

Qualitative data will be extracted and analysed following the Guidance on the Conduct of Narrative Synthesis in Systematic Reviews ( Popay *et al*. 2006): Element 1 is the role of theory in evidence synthesis; Element 2 is the development of a preliminary synthesis; Element 3 is exploring relationships within and between studies; Element 4 is the assessment of the robustness of the synthesis.

In this update, we aim to extract frameworks from the studies. Frameworks are graphical or narrative representations of a phenomenon’s factors, concepts, or variables. Implementation frameworks provide a structure for: (a) describing/guiding the process of translating effective interventions and research evidence into practice (process frameworks); (b) analysing what influences implementation outcomes (determinant frameworks); and (c) evaluating implementation efforts (outcome frameworks) ( Moullin *et al*. 2020; Nelson *et al*. 2020; Van Der Wees *et al*. 2019). The application of conceptual and theoretical frameworks is key to advancing implementation knowledge. These studies will guide clinical teams on how best to implement patient-centred outcome measures in their respective contexts by providing a foundation on generalisable evidence. The main strength of frameworks is that they organise, explain, or describe information and the range and relationships between concepts. They delineate processes and hence, are useful for communication ( Bradshaw *et al*. 2021a, 2021b). We will determine which implementation frameworks are used (if any) and how useful they are. Suboptimal use of frameworks is known to impact the viability and success of implementation efforts, resulting in wasted resources, erroneous conclusions, and specification errors in implementation methods and data analyses ( Stover *et al*. 2021).

We will consider and, where feasible, extract the PROGRESS plus ( O'Neill *et al*. 2014) characteristics: the place of residence, race/ethnicity/culture/language, occupation, gender/sex, religion, education, socioeconomic status and social capital.

### Data synthesis for implementation models and processes

We will adopt a well-tested multilevel framework for health program evaluation to describe and analyse implementation with six domains: delivery, impact, sustainability, capacity building, generalizability, and dissemination ( Masso *et al*. 2017). The extent to which this will be possible will depend on the degree of implementation undertaken and reported, including the complexity, description of constructs, visual representation and organisation levels.

We will update the recommendations on implementing patient-centred outcome measures in palliative care clinical practice generated in the original systematic review.

***Assessment of robustness and relevance of included studies***

The previous review used the Modified Harden criteria ( Barnett-Page and Thomas 2009; Slort *et al*. 2011) to appraise the quality of included studies. For this update, we will use Gough’s Weight of Evidence Framework ( Gough 2007) to assess the robustness and relevance of studies in line with our objectives; the framework applies to quantitative, qualitative and mixed-methods research at the study level. The weight of evidence refers to the preponderance of evidence used to inform decision-making. It considers judgements on different generic judgement (Weight of Evidence A), review-specific judgement on research design (Weight of Evidence B), and a review-specific criterion of evidence focus (Weight of Evidence C), and then a combination of them for an overall judgement (Weight of Evidence D) ( Gough 2004):

Weight of Evidence A (WoE A): A generic and non-review-specific judgement about the coherence and integrity of the evidence in its own terms, i.e. the generally accepted criteria for evaluating the quality of this type of evidence.

Weight of Evidence B (WoE B): A review-specific judgement about the appropriateness of that form of evidence for answering the review questions, i.e. the fitness for the purpose of that form of evidence.

Weight of Evidence C (WoE C): A review-specific judgement about the relevance of the focus of the evidence for the review question, e.g. the relevance of the type of sample, evidence gathering or analysis in relation to the review question.

These three sets of judgements can then be combined to form an overall assessment Weight of Evidence D (WoE D) of the extent that a study contributes evidence to answer a review question.

Two reviewers will independently make the assessments, with any disagreement resolved by discussion and, if needed, by a third assessor.

## Changes from the original review

We have replaced the terminology patient-reported outcome measures (PROMs) with patient-centred outcome measures (PCOMs).

Four new objectives in this update were not part of the previous review: implementation outcomes, costs, implementation models, and updating the recommendations. As a result of adding an objective to extract implementation models, we may have enough information to report on implementation processes using the multilevel framework for health program evaluation ( Masso *et al.* 2017).

We will not have any date limitations in this update.

Since more studies today use the term “implementation”, we added implementation and related terms to the search strategy after consultation and testing the search.

## Dissemination

The findings will be published in a peer-reviewed MEDLINE-indexed journal. An abstract from the current work has been accepted for EAPC 2023 (European Association for Palliative Care's 18 ^th^ World Congress) ( Shokraneh *et al.* 2023b).

### Study status

The search stage has been completed and the reviewers are screening the search results.

### Limitations

One limitation of this update is that we will focus on the settings in which palliative and end-of-life care occur. We will not be able to include studies focusing on particular conditions, such as advanced cancer, outside a palliative care context.

## Data Availability

No underlying data are associated with this article. Open Science Framework: IMPCOM: Implementing Patient-Centred Outcome Measures in Palliative Care Clinical Practice for Adults: An Update Systematic Review of Facilitators and Barriers. https://doi.org/10.17605/OSF.IO/M4F6A ( Shokraneh *et al.* 2023a). This project contains the following extended data:
‐ Appendix 1. Search Strategy for MEDLINE via Ovid SP.docx Open Science Framework: PRISMA-P checklist for ‘Implementing patient-centred outcome measures in palliative care clinical practice for adults: (IMPCOM): Protocol for an update systematic review of facilitators and barriers’. https://doi.org/10.17605/OSF.IO/M4F6A ( Shokraneh *et al.* 2023a). Data are available under the terms of the Creative Commons Zero “No rights reserved” data waiver (CC0 1.0 Public domain dedication).
