# Supplementary material for: Implementing patient-centred outcome measures in palliative care clinical practice for adults (IMPCOM): Protocol for an update systematic review of facilitators and barriers
Source: F1000Res. 2023 Oct 30;12:224. Originally published 2023 Feb 28. [Version 2] doi: 10.12688/f1000research.131479.2 (PMC10628357; doi:10.12688/f1000research.131479.2)
Supplement: Peer review history

# Reviewer report: Reviewer response for version 2

**DOI:** 10.5256/f1000research.158181.r219339

**Published:** 2023-11-06

**Authors:** Madeleine Hinwood

**Affiliations:** 1 The University of Newcastle, Callaghan, NSW, Australia; 2 Data Sciences, Hunter Medical Research Institute, New Lambton Heights, NSW, Australia

Thank you for addressing my comments.

Is the study design appropriate for the research question?

Yes

Is the rationale for, and objectives of, the study clearly described?

Yes

Are sufficient details of the methods provided to allow replication by others?

Yes

Are the datasets clearly presented in a useable and accessible format?

Not applicable

Reviewer Expertise:

Epidemiology, biostatistics

I confirm that I have read this submission and believe that I have an appropriate level of expertise to confirm that it is of an acceptable scientific standard.

# Reviewer report: Reviewer response for version 2

**DOI:** 10.5256/f1000research.158181.r219338

**Published:** 2023-10-31

**Authors:** Mitra Tewes

**Affiliations:** 1 Department of Palliative Medicine, University Hospital Essen, University of Duisburg-Essen, Essen, Germany

I confirm my 'approved' status.

Is the study design appropriate for the research question?

Yes

Is the rationale for, and objectives of, the study clearly described?

Yes

Are sufficient details of the methods provided to allow replication by others?

Yes

Are the datasets clearly presented in a useable and accessible format?

Yes

Reviewer Expertise:

Palliative Care, patient reported outcome measurement

I confirm that I have read this submission and believe that I have an appropriate level of expertise to confirm that it is of an acceptable scientific standard.

# Reviewer report: Reviewer response for version 1

**DOI:** 10.5256/f1000research.144323.r215104

**Published:** 2023-10-25

**Authors:** Mitra Tewes

**Affiliations:** 1 Department of Palliative Medicine, University Hospital Essen, University of Duisburg-Essen, Essen, Germany

The authors of the presented manuscript describe a study protocol for a systematic review of implementing patient-centered (or patient-reported) outcome measures in palliative and end-of-life care. The study objective is the identification of new evidence on facilitators, barriers, lessons learned, used instruments, models of implementation, implementation outcomes, costs, and conseqences of implementing PCOMs. Therfore, the authors describe the search tools, types of studies, participants and settings. Proxy outcome measures will be considered in the presented review to include patients in bad conditions. The study protocol describes the exclusion criteria and search methods in detail.

 The description of the underlying disease (e.g. cancer vs non-cancer) would be helpful in the "Data extraction" section.

 The changes from the original systematic review are clearly described and relevant.

 In sum, the authors deal with an important update of the systamatic review for PROMs in palliative care. Therefore, I would accept this manuscript for indexing with only the described little minor revision.

Is the study design appropriate for the research question?

Yes

Is the rationale for, and objectives of, the study clearly described?

Yes

Are sufficient details of the methods provided to allow replication by others?

Yes

Are the datasets clearly presented in a useable and accessible format?

Yes

Reviewer Expertise:

Palliative Care, patient reported outcome measurement

I confirm that I have read this submission and believe that I have an appropriate level of expertise to confirm that it is of an acceptable scientific standard.

# Reviewer report: Reviewer response for version 1

**DOI:** 10.5256/f1000research.144323.r211225

**Published:** 2023-10-06

**Authors:** Madeleine Hinwood

**Affiliations:** 1 The University of Newcastle, Callaghan, NSW, Australia; 2 Data Sciences, Hunter Medical Research Institute, New Lambton Heights, NSW, Australia

This is a protocol describing a planned update of a systematic review. The justification for updating the review is sound, and the objectives are clear.

 In the Design section of the methods, four PRISMA reporting guidelines are referenced, including PRISMA-P (for systematic review protocols). I would imagine that this protocol should be reported according to PRISMA-P, with the review itself reported according to PRISMA and PRISMA-S.

 Other changes from the previous review were reported, however not consistently. I would like to understand which things changed, and also which remained the same. For example, in 'Data synthesis', you are using an implementation framework published after the original review. What did you previously use to guide data synthesis? The contrast would be helpful here.

 Overall, this will be an interesting update, which has been well-justified and well described.

Is the study design appropriate for the research question?

Yes

Is the rationale for, and objectives of, the study clearly described?

Yes

Are sufficient details of the methods provided to allow replication by others?

Yes

Are the datasets clearly presented in a useable and accessible format?

Not applicable

Reviewer Expertise:

Epidemiology, biostatistics

I confirm that I have read this submission and believe that I have an appropriate level of expertise to confirm that it is of an acceptable scientific standard, however I have significant reservations, as outlined above.
